# Liraglutide Modulates Zinc Release and Improves Mitochondrial Function in Insulin-Resistant Senescent Cardiomyocytes

**DOI:** 10.1007/s12012-026-10095-x

**Published:** 2026-01-28

**Authors:** Fatemeh Hosseinpourshirazi, Umur D. Mendes, Zeynep B. Aksoy, Dunya Aydos, Merve Sözer, Lubne Aljaser, Manolya Gundogdu, Suatnur Sık, Erkan Tuncay, Belma Turan, Yusuf Olgar

**Affiliations:** 1 Stem Cell Institute, Ankara University, Ankara, Turkey; 2 Faculty of Pharmacy, Department of Pharmacology, Ankara University, Ankara, Turkey; 3 Interdisciplinary Food, Metabolism and Clinical Nutrition Department, Ankara University, Ankara, Turkey; 4 Faculty of Medicine, Department of Biophysics, Ankara University, Ankara, Turkey; 5 Faculty of Medicine, Department of Biophysics, Lokman Hekim University, Ankara, Turkey

**Keywords:** Liraglutide, Zinc homeostasis, Mitochondrial function, Insulin resistance, Senescent cardiomyocytes, Casein kinase

## Abstract

Aging and insulin resistance are intertwined factors in the development of metabolic diseases such as type 2 diabetes and cardiovascular disorders. Liraglutide, a glucagon-like peptide-1 (GLP-1) receptor agonist, has shown promising cardioprotective effects in preclinical and clinical studies of metabolic diseases. Yet, its action on insulin-resistant aged subjects is not clearly defined. This study aimed to investigate the effects of liraglutide on intracellular zinc levels, including its modulation of oxidative stress, mitochondrial function, and Endoplasmic Reticulum (ER) stress in a novel insulin-resistant senescent model. Insulin resistance and senescence were confirmed by reduced glucose uptake and increased β-Galactosidase Staining and increased p-H2A.X (Ser139) levels after 24 h of co-incubation with bovine serum albumin (BSA) conjugated palmitic acid (PA; 50 µM) and 278 mM D-galactose (D-Gal) in human AC16 cells. Our findings showed upregulated expression of ER and mitochondrial proteostasis markers in the early minutes of liraglutide treatment. In addition, chronic but not acute liraglutide treatment significantly increased intracellular zinc levels, accompanied by improved mitochondrial membrane potential and reduced reactive oxygen species in the insulin-resistant senescent model. Casein kinase 2 inhibition completely abolished liraglutide-induced zinc elevation and mitochondrial improvements in the chronic context, highlighting the role of casein kinase 2 in the subcellular signaling of liraglutide. These findings indicate that liraglutide alters intracellular zinc and modulates endoplasmic reticulum–mitochondria communication, giving insight into its therapeutic potential in metabolic cardiomyopathies linked to insulin resistance and aging.

**Supplementary Information:**

The online version contains supplementary material available at 10.1007/s12012-026-10095-x.

## Introduction

Aging and insulin resistance are two interconnected factors that have a key role in the development of metabolic diseases such as type 2 diabetes and cardiovascular disorders [1, 2]. Both processes are characterized by the accumulation of oxidative stress, mitochondrial dysfunction, and cellular senescence, all of which worsen the pathophysiological consequences of these factors [3–5]. Insulin resistance, defined as impaired insulin signaling, causes high blood glucose levels and contributes to the development of various diseases associated with aging [6]. Similarly, aging causes a cascade of cellular stress responses, including mitochondrial malfunction and endoplasmic reticulum (ER) stress, which impair cellular function and resilience [7, 8]. Addressing these stress pathways raises the possibility of a treatment strategy for metabolic diseases associated with aging.

Liraglutide, an agonist of the glucagon-like peptide-1 receptor, is widely employed for managing type 2 diabetes. Liraglutide has been demonstrated in recent studies to have substantial neuroprotective and cardioprotective benefits in addition to its glucose-lowering effects [9, 10]. This has sparked interest in its ability to control cellular stress reactions, especially in insulin resistance and aging models. Despite being investigated across various settings [11–13], how exactly it works to reverse mitochondrial dysfunction and control stress signaling pathways in insulin-resistant senescent subjects remains unclear.

Oxidative stress and dysregulated mitochondrial activity are hallmarks of insulin resistance and aging [14–16]. Impaired redox regulation and oxidative stress play a crucial role in increasing mitochondrial damage and interfering with insulin signaling [17]. Furthermore, the accumulation of reactive oxygen species (ROS) triggers several stress mechanisms, including the unfolded protein response, which results in ER stress and cellular malfunction [18]. In addition, mitochondrial dysfunction, which is characterized by low mitochondrial membrane potential (MMP), altered mitochondrial dynamics, and decreased mitochondrial biogenesis, contributes to metabolic failure and aging [19, 20].

A vital metal, zinc, governs many enzymatic processes and contributes to redox equilibrium [21]. Intracellular zinc ([Zn²⁺]_i_) dysregulation has been linked to many illnesses, including insulin resistance and aging [22–24]. Previous studies have demonstrated that elevated [Zn²⁺]_i_ alters ATP production [25], fosters mitochondrial quality control mechanisms [26, 27], and leads to apoptosis in a variety of cell types [28]. Yet, [Zn²⁺]_i_ is also essential for maintaining cellular homeostasis since it regulates the expression of proteins that bind metals and are rich in cysteines, like metallothioneins. These proteins are potent cytoprotectants and electrophilic scavengers that effectively regulate the balance of zinc-related cellular responses and reduce oxidative damage [29–31].

This study aimed to investigate the effects of liraglutide on the [Zn²⁺]_i_ level in a novel insulin-resistant senescent model induced by co-incubation of PA and D-Gal, where it modulates oxidative stress, mitochondrial function, and ER stress. Our findings provide insights into possible mechanisms through which liraglutide may influence cellular responses under insulin-resistant and aging-related conditions in a time-dependent manner. In addition, the data suggest that CK2 inhibition could modulate these pathways, contributing to an improved understanding of therapeutic strategies for age-associated metabolic alterations.

## Materials and Methods

### AC16 Human Cardiomyocyte Cells

AC16 cells were cultured under specific conditions to maintain their proliferative capacity and cardiomyocyte-like properties. Cells are typically grown in Dulbecco’s Modified Eagle Medium (DMEM) supplemented with 10% fetal bovine serum (FBS), 1% penicillin-streptomycin, and L-glutamine at 37 °C in a humidified atmosphere of 5% CO₂ [32]. To ensure healthy cell growth, the medium was changed every 2–3 days, and cells were passaged at approximately 80% confluence using 0.05% trypsin-EDTA. Insulin resistance and senescence were induced using BSA-conjugated PA with a final PA: BSA molar ratio of ~ 17:1, applied at a final concentration of 50 µM PA (corresponding to ~ 3 µM BSA). D-Gal was applied at a final concentration of 278 mM.

### Glucose Uptake Assay

Cellular glucose uptake was assessed using the Invitrogen 2-NBDG kit (Item No. N13195), which utilizes the fluorescent glucose analog 2-NBDG as previously employed [33]. Briefly, cells were washed with PBS, mounted in a recording chamber, and incubated with 100 µM 2-NBDG in the glucose-free medium. Fluorescence signals (excitation: 488 nm, emission: 525 nm) were recorded using time-lapse acquisition on a confocal microscope (Leica-SP5). Resting glucose uptake was measured for 60 min, while insulin-dependent uptake cells were monitored after incubation with 100 nM insulin, followed by an additional 1-hour recording. Glucose uptake was quantified by region of interest (ROI) analysis, with mean fluorescence intensity measured for manually defined ROIs around individual cells. The images were analyzed using LASX (Leica Systems) software.

### Reactive Oxygen Species Measurements

ROS levels in AC16 human cells were assessed using the fluorescent probe DCFH-DA. DCFH-DA enters cells, where it is hydrolyzed to DCFH and then oxidized by ROS to form the fluorescent DCF. The cells were incubated with 10 µM DCFH-DA for 45 min and shielded from light to minimize photobleaching. After removing the excessive dye, ROS levels were measured using a confocal microscope (Leica-SP5), with fluorescence excited at 485 nm and emitted at 530 nm. Fluorescence measurements were performed by selecting regions of interest (ROIs) within individual cells to standardize the analysis and avoid bias from varying cell counts across images. The accuracy of the ROS assay was confirmed by calibrating the measurements with a 100 µM hydrogen peroxide (H₂O₂) treatment, as described elsewhere [34, 35]. All fluorescence measurements were quantified using ROI labeling. For each image, ROIs were manually defined around individual cells, and the mean fluorescence intensity within each ROI was measured. The images were analyzed using LASX software.

### Mitochondrial Membrane Potential Measurements

The mitochondrial membrane potential in AC16 human cardiomyocytes was measured with a fluorescence-based assay as described elsewhere [35]. Cells loaded with the membrane-permeant dye JC-1 (5-µmol/L, 30 min, 37 °C) and imaged with a confocal fluorescence microscope (Leica TCS SP5). The green fluorescence of JC-1 monomers was detected at an emission wavelength of 535 nm following excitation at 488 nm, while the red fluorescence from JC-1 aggregates was captured at an emission wavelength of 585 nm. Carbonyl cyanide 4-(trifluoromethoxy) phenylhydrazone (FCCP; 10 µmol/L) was used as a positive control for MMP. Fluorescence measurements were conducted using selected ROIs within individual cells to account for any variation in cell counts. The images were analyzed using LASX software.

### Measurement of Cytosolic Free Zinc Levels in AC16 Cells

To determine ([Zn^2+^]_i_), cells were incubated with 3 µM FluoZin3-AM, a dye with a high affinity for zinc (Kd = 15 nM), for 30–40 min. The dye is excited at 490 nm and emits at 520 nm. Following incubation, cells washed 4–5 times for de-esterification, preparing them for [Zn^2+^]_i_ measurement. Intracellular free zinc concentrations were measured using a confocal microscope (Leica-SP5). After recording baseline readings (F), cells were treated sequentially with the Zn^2+^ ionophore 1-Hydroxypyridine-2-thione zinc salt (ZnPT; 10 µM) to obtain the maximum signal (F_Max_), followed by the Zn^2+^ chelator Tetrakis-(2-Pyridylmethyl)ethylenediamine (TPEN; 50 µM) to obtain the minimum signal (F_Min_). Using the known Kd value of FluoZin-3, basal zinc concentration was calculated using the equation: [Zn^2+^]_i_=Kd×(F_Max_−F)(F − F_Min_). Fluorescence quantification was performed at the single-cell level using ROI analysis, minimizing the influence of cell density differences across images. The images were analyzed using LASX software.

### Reverse Transcription-Quantitative PCR (RT-qPCR)

To assess mRNA levels, total RNA was extracted from the AC16 cells using the PureZOL™ RNA Isolation Reagent (7326880, Bio-Rad). RNA quality and concentration were assessed by electrophoresis on 1.5% Agarose gel (Fig. S1) and by a NanoDrop spectrophotometer (Thermo Scientific) (Supplementary Table 1). 1ug of RNA was reverse transcribed using the iScript™ cDNA Synthesis Kit (1708891, Bio-Rad). Quantitative PCR (qPCR) was performed in a Roche LightCycler 480 II system with SsoAdvanced™ Universal SYBR^®^ Green Supermix (1725271, Bio-Rad). Fold changes in gene expression were determined using the comparative (2 − ΔΔCt) method, with the untreated AC16 control group serving as the calibrator and β-Actin as the housekeeping control. Primers are listed in Supplementary Table 2.

### Western Blotting

At the end of the incubations, cells were rapidly transferred to a custom-prepared lysis buffer containing NP-40 (2 M NaCl, 1 M Tris; pH 8.2, 1% NP-40, and 1X protease inhibitor cocktail). Whole-cell protein extracts were centrifuged at 13.000 × g for 20 min at 4 °C. The total protein concentration was subsequently quantified using a NanoDrop spectrophotometer (Thermo Scientific).

Western blot analysis was performed to determine the relative protein and phosphorylation levels of P62, Calnexin, LC3, ATF4, ATF6, GRP78, and DDIT3, compared with reference proteins B-actin and GAPDH. Equal amounts of protein (8–10 µg) were separated on 10–12% SDS-PAGE. Proteins were transferred (Bio-Rad – Trans-Blot Turbo) to PVDF membranes and blocked with 3% BSA for 90 min. Membranes were probed overnight with monoclonal (mono) or polyclonal (poly) primary antibodies diluted in 3% BSA for P62 (814801; 1:500; BioLegend), Calnexin (sc-23954; 1:500; Santa Cruz), LC3 (848802, 1:500, BioLegend), ATF4 (STJ11101481; 1:500; BioLegend), ATF6 (STJ114444; 1:500; St John’s Laboratory), GRP78 (sc-13968; 1:500; Santa Cruz), B-actin (664802; 1:500; BioLegend), and GAPDH (649202, 1:500; BioLegend) as loading controls. Specific bands were visualized with HRP-conjugated compatible secondary antibodies. The density of bands was analyzed using ImageJ software [35, 36].

### p-H2A.X (Ser139) Immunofluorescence Staining

Phosphorylated H2A.X (p-H2A.X, Ser139) expression in AC-16 cells was evaluated by immunofluorescence staining using confocal microscopy [36]. Following the experimental treatments, cells were fixed with 4% paraformaldehyde and permeabilized using 0.3% Triton X-100. Immunostaining was performed using a primary antibody against p-H2A.X (Thermo, MA5-14957; Ser139), while β-actin (Santa Cruz, sc-47778) was used as a cytoskeletal marker to visualize cellular structure. After overnight incubation with primary antibodies at 4 °C, cells were incubated with appropriate fluorescently labeled secondary antibodies diluted in 0.25% Triton X-100/PBS. Nuclei were counterstained with DAPI, and coverslips were mounted using an antifade mounting medium. Fluorescent images were acquired under identical imaging conditions for all groups, and p-H2A.X staining was used as an indicator of DNA damage–associated senescence.

### Statistical Analysis

Data were tested for normality using the Shapiro-Wilk test. For non-parametric data, differences between groups were analyzed using the Kruskal-Wallis test. Post-hoc comparisons were performed using Dunn’s test with Bonferroni correction to account for multiple comparisons and minimize the risk of Type I errors, particularly given the variation in sample sizes. This correction ensures that statistical significance is appropriately controlled despite the unequal number of data points between groups. Statistical analysis is presented as mean ± standard error of the mean (SEM), *p* < 0.05 considered significant.

## Results

### Characterization of Insulin-Resistant-Senescence Models

**Fig. 1:**
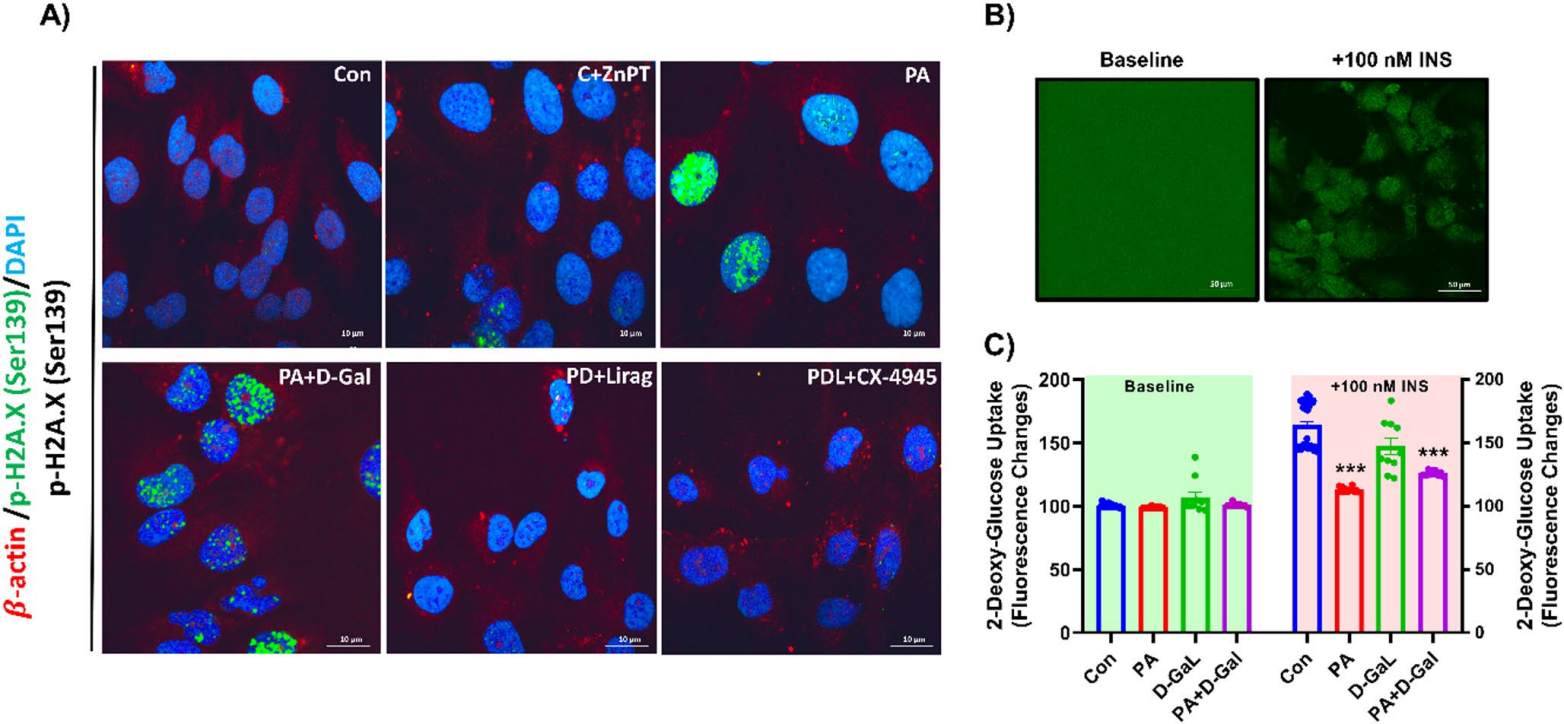
Characterization of the insulin-resistant aging model and its physiological modulation by liraglutide and CK2 inhibition in AC-16 cells. **A** Representative immunofluorescence images showing p-H2A.X (Ser139) expression (green), β-actin (red), and DAPI nuclear staining (blue) in experimental groups. Scale bar is 10 μm. **B** Glucose Uptake Assay with 2-Deoxyglucose (2-DG) fluorescence uptake in AC-16 cells. Representative fluorescence images show 2-DG uptake in cells under insulinotropic conditions. The fluorescence intensity corresponds to intracellular glucose uptake levels. Scale bar is 50 μm. **C** Quantitative analysis of glucose uptake under insulinotropic stimulation reveals a significant reduction in the PA and P + D-Gal groups compared to the Con group. Results are expressed as mean ± SEM, with the number of analyzed cells; n_(Con)_:25, n_(PA)_: 10, n_(D-Gal)_:10 and n_(P+D-Gal)_:12. Statistical analysis was performed using the Kruskal-Wallis test, with ***p < 0.001 versus Con.

Cell viability was evaluated using the XTT assay before conducting other experiments. Incubation of BSA conjugated PA (50 µM) and P + D-Gal (278 mM D-galactose) for 24 h showed no effects on cell viability or metabolic function (Supplementary Fig. 1). Microscopic analysis revealed increased blue staining in the PA-treated group, with a more pronounced intensity in the P + D-Gal group, indicating the induction of cellular senescence. Notably, treatment with liraglutide and CK2 inhibition markedly attenuated this senescence-associated blue staining (Supplementary Fig. 2). These observations were further supported by an increase in p-H2A.X (Ser139) levels in PA, P + D-Gal and external Zinc (C + ZnPT, 10 nM) groups, a well-established marker of DNA double-strand breaks [37], indicating a decrease in DNA damage–associated senescence and supporting the protective effects of liraglutide treatment and CK2 inhibition (Fig. 1A). Insulin resistance was confirmed using glucose uptake assays under insulin-dependent challenge. A significant decrease in insulin-stimulated glucose uptake was observed in groups treated with PA and P + D-Gal for 24 h, indicating the establishment of insulin resistance (Fig. 1B).

Taken together, these results suggest that co-incubation with PA and D-Gal at the specified concentrations is associated with the induction of senescence and insulin resistance, thereby providing a suitable in vitro model for exploring cellular pathways related to insulin resistance and aging, and for assessing the potential modulatory effects of liraglutide.

### Bimodal Effects of Liraglutide on ROS and Mitochondrial Function in Insulin-Resistant Senescence Model

**Fig. 2:**
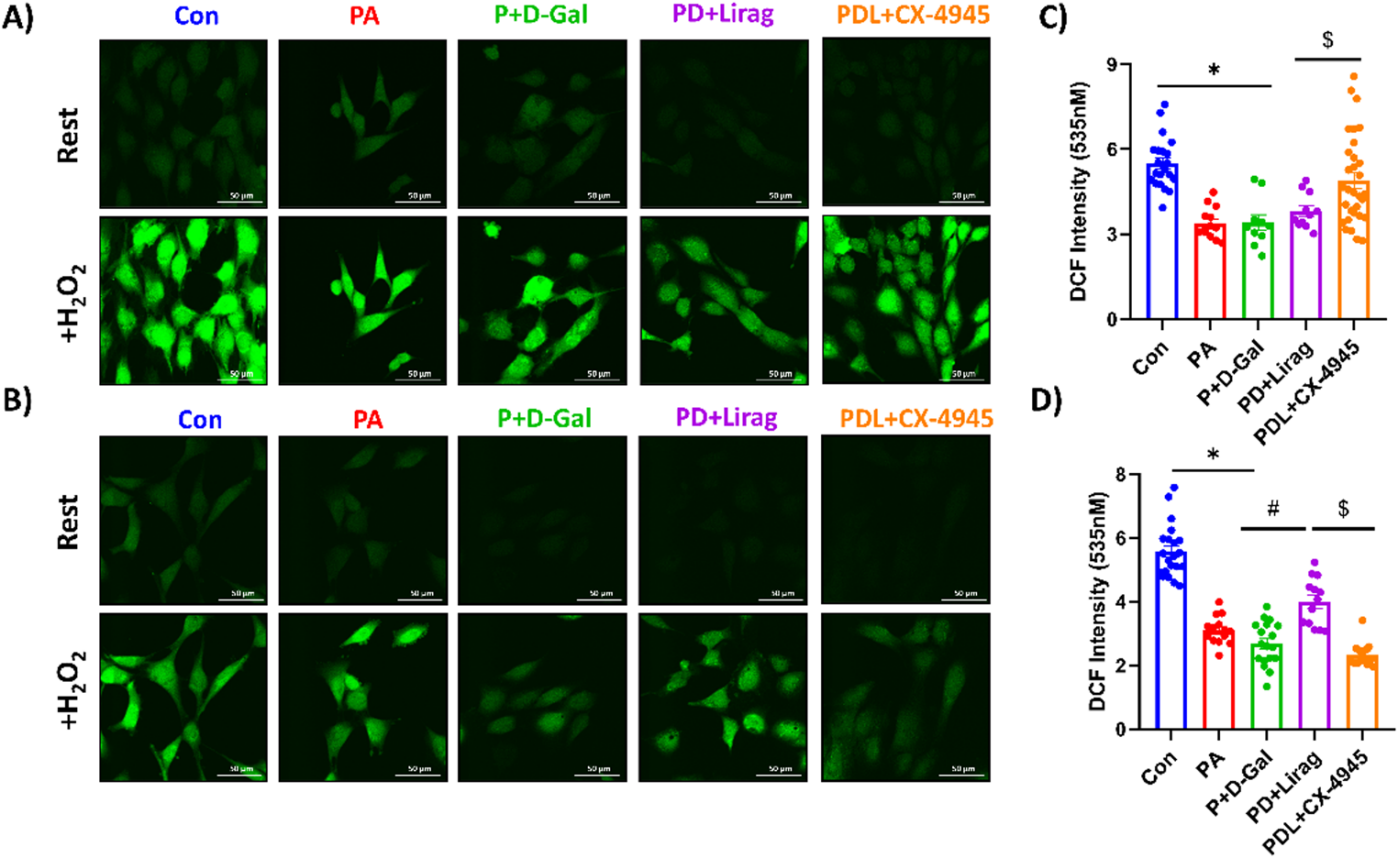
Measurement of ROS levels using DCFDA fluorescence staining in experimental groups. AC-16 cells were incubated with DCFDA (5 µM) for 30 min at 37 °C to assess intracellular ROS levels. ROS production was quantified by evaluating fluorescence intensity following treatment with 100 µM H₂O₂, where higher fluorescence intensity indicates lower ROS levels at resting conditions. Representative images illustrate ROS levels under 30-minute acute (**A**) and 6-hour chronic (**B**) liraglutide and CX-4945 treatments, with summary data plotted in panels (**C**, **D**), respectively. Results are expressed as mean ± SEM, with the number of cells analyzed; n_(Con)_:22 − 21, n_(PA)_: 13–14, n_(P+D-Gal)_:10–18, n_(PD+Lirag)_:12, and n_(PDL+CX-4945)_:31 − 14. Statistical analysis was performed using the Kruskal-Wallis test, with *p < 0.01 versus Con, ^#^*p* < 0.05 versus P + D-Gal, and ^$^*p* < 0.05 vs. Pd + Lirag. Scale bar is 50 μm.

The cellular damage associated with insulin resistance is largely caused by increased oxidative stress, which is typified by higher levels of reactive oxygen species. Acute incubation with liraglutide (1 µM) for 30 min did not significantly affect the increased ROS formation in the P + D-Gal group whereas treatment with the CK2 inhibitor, CX-4945 (5 µM), significantly reduced ROS levels, indicating a potential antioxidant effect mediated by CK2 inhibition (Fig. 2C). Chronic treatment with liraglutide (6-h) markedly yielded a reduction in ROS levels in P + D-Gal groups, whereas CK2 inhibition resulted in a significant increase in ROS, illustrating two opposing effects of liraglutide and CK2 inhibition over time in insulin-resistant aged cells (Fig. 2D).

**Fig. 3:**
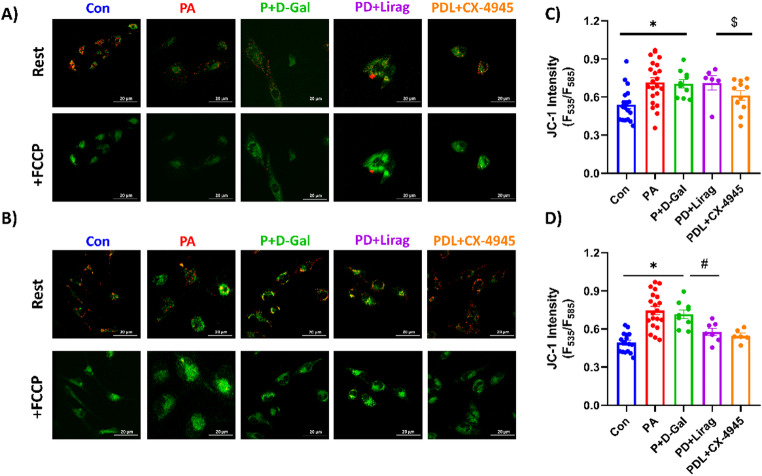
Assessment of MMP using JC-1 fluorescence staining in experimental groups. AC-16 cells were incubated with 5 µM JC-1 for 30 min at 37 °C. MMP was assessed by the red-to-green fluorescence ratio, with higher ratios indicating more polarized mitochondria. Representative images show MMP levels under 30-minute acute (**A**) and 6-hour chronic (**B**) liraglutide and CX-4945 treatments, with summary data presented in panels **C**, **D**, respectively. Results are shown as mean ± SEM, with number of the analyzed cells: n_(Con)_:19–19, n_(PA)_: 25 − 24, n_(P+D-Gal)_:10–10, n_(PD+Lirag)_:6–7 and n_(PDL+CX-4945)_:11 − 6. Statistical analysis was performed using the Kruskal-Wallis test, with *p < 0.01 vs. Con, ^#^*p* < 0.05 versus P + D-Gal and ^$^*p* < 0.05 vs. PD-Lirag. The Scale bar is 20 μm.

JC-1 staining revealed a substantial depolarization of MMP in the P + D-Gal group in comparison to controls. Acute treatment with liraglutide did not significantly affect the depolarized MMP, whereas CK2 inhibition with CX-4945 significantly improved MMP (Fig. 3C). In contrast, chronic treatment with liraglutide alone significantly improved MMP in the P + D-Gal group, without any further impact from CK2 inhibition (Fig. 3D). These results suggest that CK2 inhibition can provide acute improvements in mitochondrial function, while chronic liraglutide treatment leads to long-term effects in mitochondrial health in the insulin-resistant aged model.

### Liraglutide Modulates Cytosolic Free Zinc Levels in Insulin-Resistant Senescence Models

**Fig. 4:**
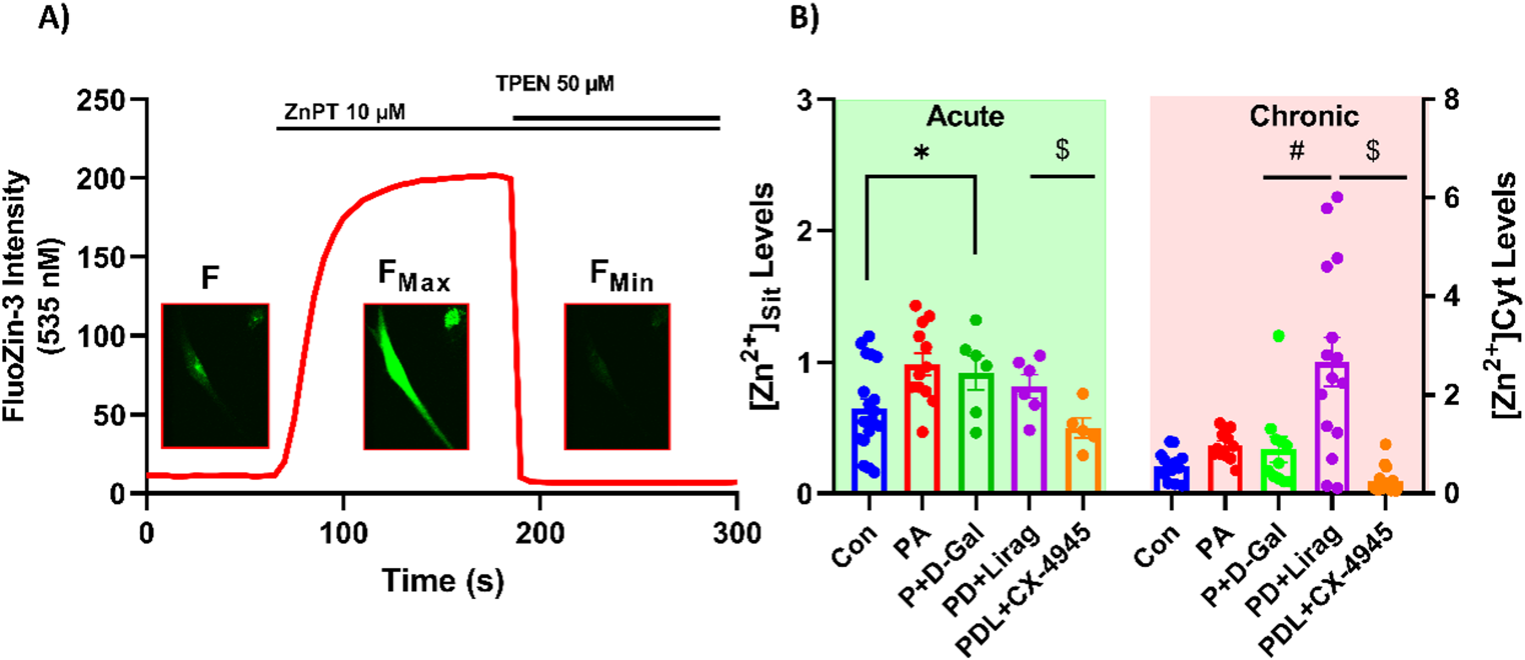
Assessment of cytosolic free zinc levels using FluoZin-3 AM fluorescence staining in experimental groups. AC-16 cells were incubated with Fluo-3 AM (5 µM) for 30 min at 37 °C to evaluate [Zn²⁺]_i_. **A** Cytosolic free zinc levels were calculated using the equation: ***[Zn²⁺]i=Kd×(F − F***_***Min***_***)/(F***_***Max***_***−F)***, where K_d_ = 15 nM. F_Max_ was determined using 10 µM ZnPT, and F_Min_ with 50 µM TPEN. **B** Calculated [Zn²⁺]_i_ levels for 30-minute acute and 6-hour chronic liraglutide and CX-4945 treatments in insulin-resistant aged cells are shown. Results are given as mean ± SEM, n_(Con)_:20 − 16, n_(PA)_: 12–12, n_(P+D-Gal)_:6–11, n_(PD+Lirag)_:6–16 and n_(PDL+CX-4945)_:5–17 number of cells analyzed per group. Statistical analysis was performed using the Kruskal-Wallis test, with *p < 0.05 versus Con, ^#^*p* < 0.05 versus P + D-Gal and ^$^*p* < 0.05 vs. PD + Lirag.

Cytosolic free zinc levels [Zn²⁺]_i_ are crucial for cellular function, and their dysregulation is associated with insulin resistance and aging. In our insulin-resistant aged model, [Zn²⁺]_i_ levels were significantly elevated (Fig. 4B, Left), indicating that dysregulation of zinc homeostasis may be associated with oxidative stress. Acute treatment with liraglutide did not significantly alter the elevated zinc levels, whereas CK2 inhibition with CX-4945 reduced [Zn²⁺]_i_ levels in this group, suggesting that CK2 inhibition may counteract the zinc elevation in response to oxidative stress (Fig. 4B, Left). Chronic liraglutide treatment led to a significant increase (approx. 5-fold) in [Zn²⁺]_i_ levels compared to the P + D-Gal group (Fig. 4B, Right). CK2 inhibition resulted in marked reduction of [Zn²⁺]_i_, highlighting an opposing interactions between liraglutide and CK2 inhibition in the regulation of zinc homeostasis (Fig. 4B, Right).

### Liraglutide Modulates ER-Stress in Insulin-Resistant Senescence Models

**Fig. 5:**
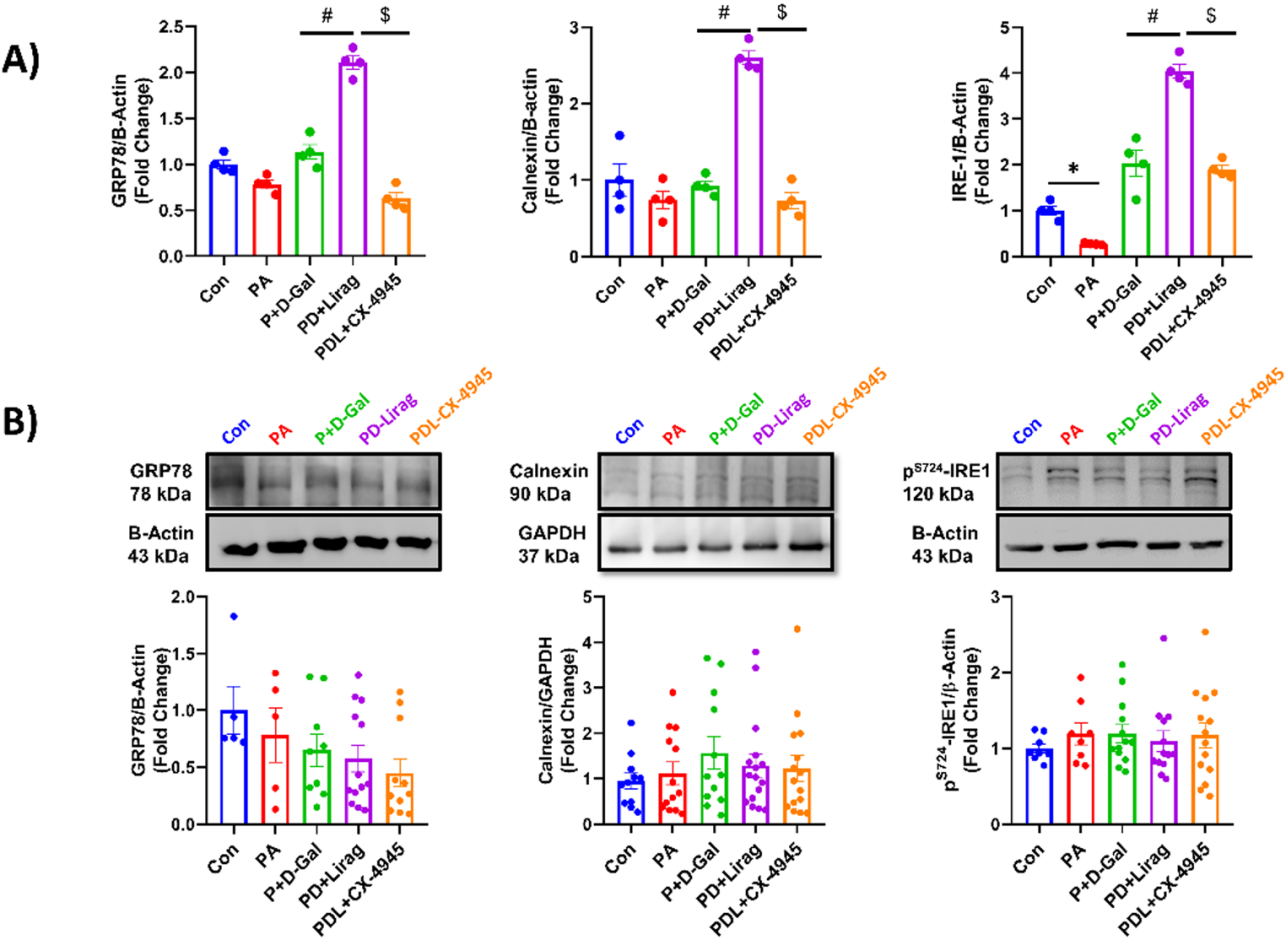
Expression levels of ER-stress markers in experimental groups. Insulin resistant AC-16 senescent cells were treated with liraglutide and CX-4945 for 30 min or 6 h for mRNA and protein expression levels, respectively. **A** mRNA expression levels of ER-stress markers and (**B**) protein levels were shown. Results were presented as fold change relative to the Con group. Results are given as mean ± SEM, n_(Con)_:4–5, n_(PA)_: 4–5, n_(P+D-Gal)_:4–9, n_(PD+Lirag)_:4–13 and n_(PDL+CX-4945)_:4–17 number of replicates. Statistical analysis was performed using the Kruskal-Wallis test, *p < 0.05 vs. Con, ^#^*p* < 0.05 vs. P + D-Gal and ^$^*p* < 0.05 vs. PD + Lirag.

ER stress is known to exacerbate insulin resistance by impairing cellular functions and promoting inflammation. Acute liraglutide treatment was associated with increased mRNA levels of the ER stress–related markers GRP78, Calnexin, and IRE1 in the PD + Lirag group, while CK2 inhibition attenuated these liraglutide-associated increases, suggesting a potential involvement of CK2 in the modulation of ER stress–related responses (Fig. 5A). Interestingly, no significant changes were observed among the groups under chronic conditions (Fig. 5B).

**Fig. 6:**
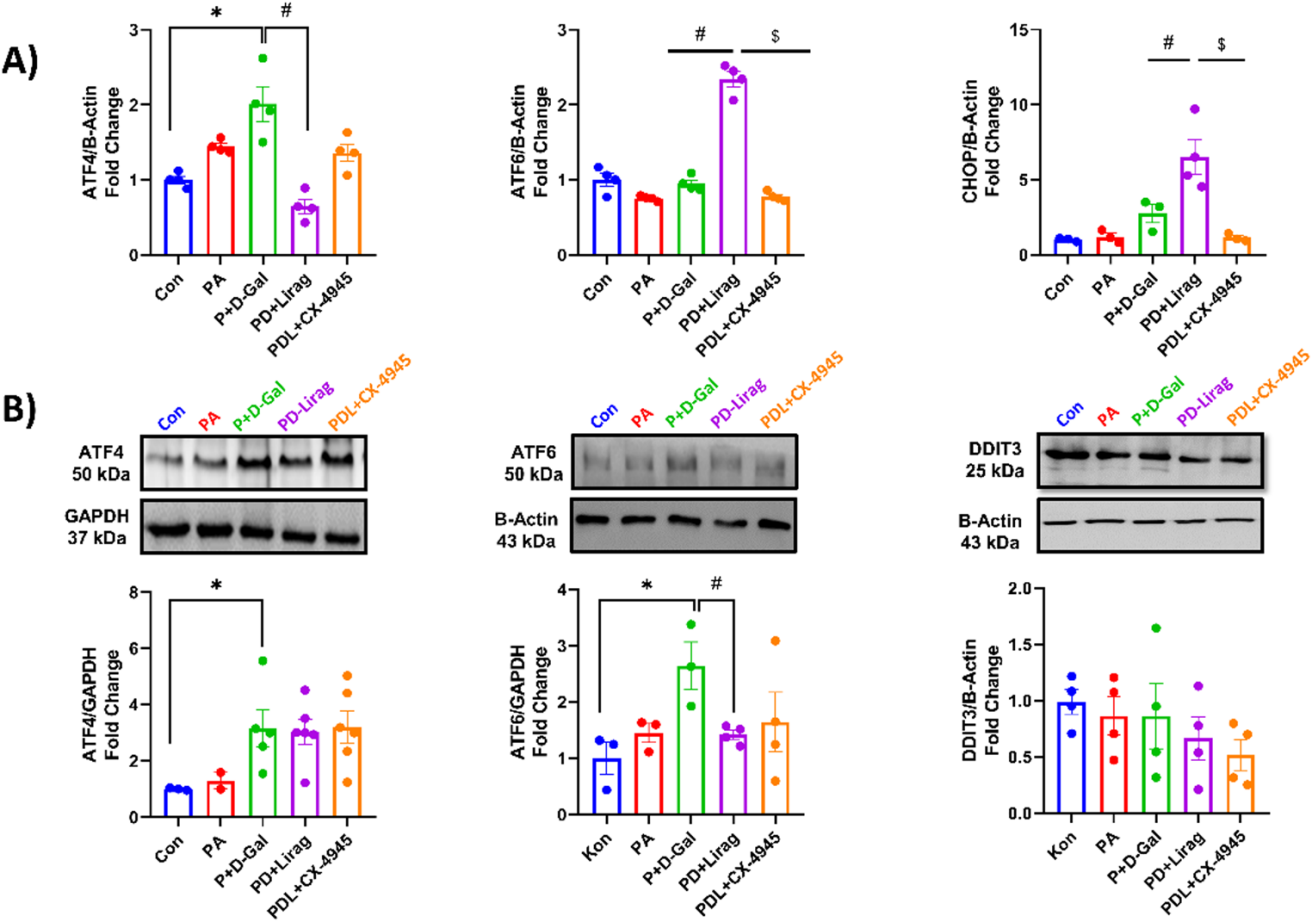
Expression levels of integrated stress markers ATF4, ATF6, and CHOP in insulin-resistant aged cells. **A** Relative mRNA expression levels of ER stress markers ATF4, ATF6, and CHOP in insulin resistant senescent AC16 cells were treated with Liraglutide and CX-4945 for 30 min. Data are normalized to B-Actin mRNA levels and expressed as fold changes relative to the Con group. Results are given as mean ± SEM, n_(Con)_:4, n_(PA)_: 4, n_(P+D-Gal)_:4, n_(PD+Lirag)_:4 and n_(PDL+CX-4945)_:4 number of replicates. **B** Representative western blot images (top) and quantitative summary data (bottom) showing ATF4, ATF6, and DDIT3 (CHOP) protein levels in the same experimental groups (*n* = 4). Results are presented as mean ± SEM. n_(Con)_:4, n_(PA)_: 4, n_(P+D-Gal)_:4, n_(PD+Lirag)_:4 and n_(PDL+CX-4945)_:4 number of replicates. Statistical comparisons were performed using the Kruskal-Wallis test. *p < 0.05 vs. Con, #*p* < 0.05 vs. P + D-Gal, $*p* < 0.05 vs. PD + Lirag.

ATF4 levels were significantly increased in the P + D-Gal group, whereas no significant changes were observed in ATF6 or CHOP levels (Fig. 6A), indicating that ER stress signaling in this model is predominantly associated with ATF4-related responses. Acute liraglutide treatment was associated with a reduction in ATF4 mRNA levels and concomitant increases in ATF6 and CHOP mRNA levels (Fig. 6A), suggesting a transient modulation of unfolded protein response–related pathways. Under chronic conditions, liraglutide treatment was associated with a reduction in the elevated ATF6 protein expression, while ATF4 protein levels remained persistently increased and CHOP expression remained unchanged (Fig. 6B).

### Liraglutide Enhances the Mitochondrial Quality Control in Insulin-Resistant Senescence Models

**Fig. 7:**
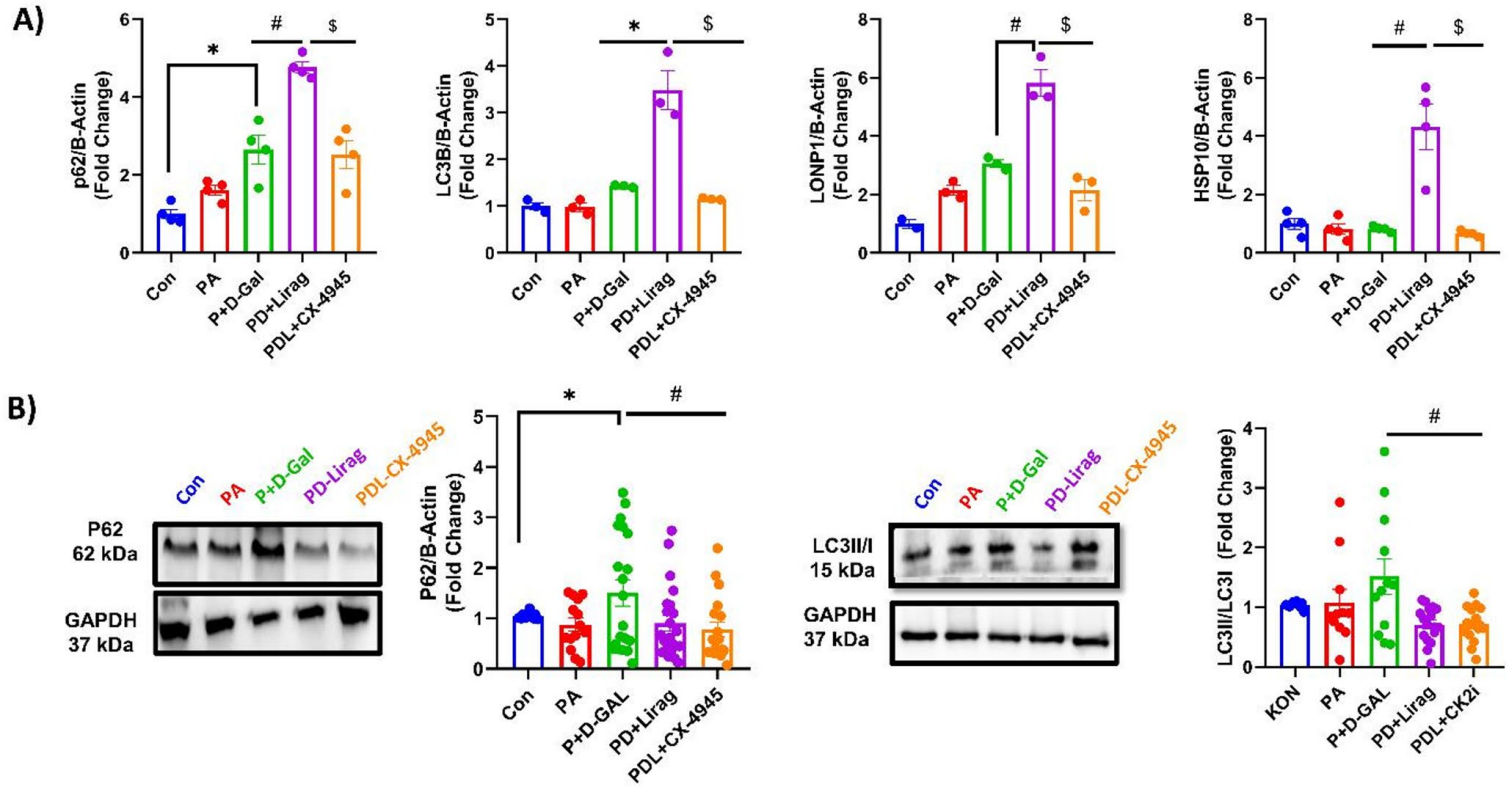
Analysis of mRNA and protein expression levels of mitochondrial proteostasis markers. **A** Relative mRNA expression levels of mitochondrial proteostasis markers p62, LC3I/II, LONP1, and HSP10 in aged AC16 cells. Data are normalized to B-Actin and mRNA levels and expressed as fold changes relative to the Con group. Results are presented as mean ± SEM, n_(Con)_:4, n_(PA)_: 4, n_(P+D-Gal)_:4, n_(PD+Lirag)_:4 and n_(PDL+CX-4945)_:4 number of replicates. **B** Representative Western blot images (Left) and calculated summary data for p62, LC3I/II protein expression in the same experimental groups. Results are presented as mean ± SEM, n_(Con)_:10 − 5, n_(PA)_: 10 − 5, n_(P+D-Gal)_:12 − 6, n_(PD+Lirag)_:21 − 14 and n_(PDL+CX-4945)_:17 − 14 number of replicates. Statistical significance was determined by the Kruskal-Wallis test. *p < 0.05 vs. Con, #*p* < 0.05 vs. P + D-Gal, $*p* < 0.05 vs. PD + Lirag.

Mitochondrial quality control plays a critical role in maintaining mitochondrial function and cellular homeostasis, particularly in the context of age-related diseases and insulin resistance. Although only p62 mRNA levels were upregulated in the P + D-Gal group, acute liraglutide incubation resulted in the upregulation of mitophagy markers p62 and LC3B, along with mitochondrial proteostasis markers LONP1 and HSP10 (Fig. 7A). Notably, CK2 inhibition completely reversed all these changes (Fig. 7A), indicating that CK2 inhibition counteracts the liraglutide-induced enhancement of mitochondrial quality control.

In the chronic setting, we evaluated the protein expression levels of mitophagy markers p62 and the LC3II/I ratio in experimental groups. Following a 6-hour liraglutide incubation, both p62 expression and the LC3II/I ratio decreased, suggesting the termination of mitophagic flux in the insulin-resistant aged group (Fig. 7B). These findings indicate a potential shift in mitophagy efficiency in response to chronic liraglutide treatment in insulin-resistant aged cells. However, CK2 inhibition did not significantly affect these mitophagy markers, suggesting that liraglutide modulates mitophagy independently of CK2 in the chronic setting.

## Discussion

The present study provides insights into the role of Liraglutide in modulating oxidative stress, mitochondrial function, and the UPR in insulin-resistant senescent models. We utilized a newly established system of insulin resistance and senescence induced by co-incubation of PA and D-Gal, which effectively mimicked the pathophysiological processes observed in aging and metabolic diseases. Our findings demonstrate that liraglutide has distinct, time-dependent effects on mitochondrial function, zinc homeostasis, and ER-stress signaling and CK2 plays a pivotal role in these processes and may serve as a key mediator in the transition from acute to chronic liraglutide responses, particularly by coordinating mitochondrial quality control and stress adaptation mechanisms.

The results from the glucose uptake assays confirmed that 24-hour co-incubation with PA and D-Gal resulted in significant insulin resistance in both insulin-dependent and insulin-independent conditions [38, 39]. This was accompanied by increased senescence, as evidenced by enhanced β-galactosidase staining and elevated p-H2A.X (Ser139) levels, a well-established marker of DNA double-strand breaks. Notably, liraglutide exerted protective effects, highlighting its potential as a promising therapeutic agent in senescence-associated dysfunctions.

Oxidative stress is a key player in the onset of insulin resistance and cellular senescence [14–16]. In our model, ROS levels were significantly elevated in both PA and P + D-Gal groups compared to control cells, confirming the presence of oxidative stress (Fig. 3B). These results are in line with earlier studies that demonstrate the role of oxidative stress in the pathophysiology of insulin resistance [17]. There is an intricate mechanism by which excessive ROS generation disrupts insulin signaling pathways and leads to aging-related metabolic dysfunctions [40]. Interestingly, while acute liraglutide treatment did not significantly modulate ROS levels in the aging, chronic liraglutide treatment significantly reduced ROS levels, providing evidence for its long-term antioxidant potential [9]. Liraglutide has been shown to reduce oxidative stress through multiple interconnected mechanisms. It suppresses ROS production by inhibiting the SR-Ca²⁺–xanthine oxidase (XO)–ROS axis via GLP-1R/PI3K/Akt signaling, thereby stabilizing intracellular Ca²⁺ homeostasis and limiting ROS generation in endothelial cells under stress conditions [41]. In addition, liraglutide attenuates ROS-mediated activation of the NLRP3 inflammasome by activating SIRT1, which leads to decreased NOX4 expression and reduced oxidative damage and inflammatory signaling in cardiomyoblasts [42]. On the other hand, CK2 inhibition with CX-4945 led to a reduction in ROS levels both acutely and chronically, suggesting a potential antioxidant effect mediated by CK2 inhibition, highlighting a possible role of zinc signaling in the regulation of cellular senescence [34, 43–45].

Mitochondrial membrane potential is a key indicator of mitochondrial health, and its depolarization is a hallmark of mitochondrial dysfunction in insulin resistance and aging [14, 19]. In our study, PA and P + D-Gal treatment significantly depolarized MMP, which were not affected by acute liraglutide treatment but were improved by CK2 inhibition. Chronic liraglutide treatment, however, improved MMP significantly, suggesting that liraglutide may help restore mitochondrial function in the long term. Previous studies have demonstrated that the GLP-1R agonist, commonly used in type 2 diabetes treatment, reverses AGEs-induced chondrocyte inflammation and apoptosis by suppressing RAGE signaling [11]. In elderly subjects, it also demonstrated cardioprotective properties by replicating the actions of antioxidants [46]. Together, these findings highlight the importance of treatment duration in determining the effectiveness of liraglutide in alleviating mitochondrial dysfunction in insulin-resistant aged models, while CK2 inhibition further underscores the beneficial role of the liraglutide–CK2 axis in enhancing mitochondrial ATP production.

Cytosolic free zinc levels are essential for cellular function, and their dysregulation is associated with insulin resistance and aging [22–24]. In our insulin-resistant aged model, we observed significantly elevated [Zn²⁺]_i_ levels, which were consistent with previous findings indicating zinc dysregulation in insulin resistance and aging [19, 39, 47]. Interestingly, acute liraglutide treatment did not significantly alter (Zn²⁺)_i_, while chronic liraglutide treatment significantly increased it (Fig. 4B). Previous studies indicated that the elevated concentration of ([Zn²⁺]_i_) depolarizes MMP [25], induces mitochondrial quality controls [26, 27], and triggers lysosomal-mitochondrial axis-mediated apoptosis in various cell types [28]. On the other hand, it also modulates the expression of metal-binding cysteine-rich proteins such as metallothioneins, thereby regulating zinc-related cell homeostasis as a powerful electrophilic scavenger and cytoprotectants [29–31]. These observations suggest that chronic liraglutide treatment may be associated with changes in cellular zinc homeostasis, potentially linked to mitochondrial quality control processes (Fig. 7), while coinciding with improved redox balance. Previous studies in Saccharomyces cerevisiae and mammalian cells have suggested a role for CK2 in the regulation of metal homeostasis [48, 49]. CK2 has been reported to regulate metal channels involved in sodium and zinc transport [43, 50, 51], suggesting a role in cellular metal homeostasis. CK2-mediated phosphorylation of zinc transport–related proteins may promote cytosolic Zn²⁺ release, which could subsequently be transported into mitochondria and influence mitochondrial function and ER stress–related signaling [52].

ER stress plays a critical role in the development of insulin resistance by impairing cellular functions and promoting inflammation [53]. In our study, acute liraglutide treatment increased the mRNA expression of ER stress markers, including GRP78, Calnexin, and IRE-1 (Fig. 5A). This suggests that liraglutide may induce an adaptive ER stress response in the context of the insulin-resistant aging model. Chronic liraglutide treatment did not significantly alter these markers, suggesting that Liraglutide’s effects on ER stress may only be more prominent acutely. This finding is in line with previous research showing that GLP-1 receptor agonists can induce ER stress to improve insulin sensitivity [54]. In this study, GLP-1 agonists directly modulate the ER stress response, at least in part, by inhibiting the mTOR signaling pathway in adipocytes. Another study suggests that liraglutide contributes to cellular resilience under stress conditions by inducing ER proteostasis and autophagy machinery homeostasis in the human SH-SY5Y neuroblastoma cells [13]. Its inhibition reversed these changes, indicating that CK2 inhibition can mitigate liraglutide-induced ER stress signaling through changing metal status within ER [48].

Mitochondrial quality control, including mitophagy and proteostasis, is crucial for maintaining mitochondrial function, particularly in the context of aging and insulin resistance [55, 56]. Our findings indicate that chronic liraglutide treatment modulates mitophagy in insulin-resistant aged cells. The decreased p62 expression and LC3II/I ratio after 6-hour liraglutide incubation suggest the termination of mitophagic flux, potentially reflecting enhanced mitochondrial turnover. Notably, CK2 inhibition had only effect on mitophagy markers in acute context, indicating that liraglutide regulates mitophagy independently of CK2 in the chronic setting. This suggests the involvement of alternative pathways, such as PINK1/Parkin or AMPK/mTOR signaling [57]. These results highlight liraglutide’s role in mitochondrial quality control and warrant further investigation into its long-term impact on metabolic health.

## Conclusion

Our findings provide insight into the potential cellular actions of liraglutide under aging-associated insulin-resistant conditions. At the molecular level, liraglutide was associated with changes in mitochondrial quality control, markers of DNA damage–related senescence, and oxidative stress, suggesting a possible improvement in mitochondrial and redox homeostasis. These effects may involve interconnected pathways related to mitochondrial Ca²⁺ and Zn²⁺ signaling, ROS dynamics, and downstream cellular stress responses. Functionally, such molecular adaptations could be associated with preserved mitochondrial membrane potential, altered ATP production, and enhanced cellular tolerance under insulin-resistant conditions. Collectively, these observations suggest that GLP-1 receptor agonists may have a role in modulating age-related metabolic alterations and insulin resistance–associated cellular dysfunction, providing a rationale for future in vivo and clinical investigations.

However, several limitations should be acknowledged. First, the study was conducted in a newly developed in vitro aging and insulin resistance model, which may not fully recapitulate the complexity of in vivo metabolic and inflammatory interactions. Second, the duration of treatment protocols was restricted, and the lack of additional selective mitophagy markers limited our ability to comprehensively assess mitophagic flux. Despite these limitations, the present findings hold clinical relevance, as they support the concept that liraglutide may confer benefits beyond glycemic control by targeting mitochondrial dysfunction and senescence-related pathways.

## Supplementary Information

Below is the link to the electronic supplementary material.

Supplementary Material 1

## Data Availability

Data are available from the authors upon request.
