# Supplementary material for: Liraglutide Modulates Zinc Release and Improves Mitochondrial Function in Insulin-Resistant Senescent Cardiomyocytes
Source: Cardiovasc Toxicol. 2026 Jan 28;26(2):22. doi: 10.1007/s12012-026-10095-x (PMC12852161; doi:10.1007/s12012-026-10095-x)
Supplement: Supplementary file 1 — Supplementary Material 1 [file 12012_2026_10095_MOESM1_ESM.docx]

**XTT Assay for Cell Viability**

Cells were seeded in 96-well plates at 1x10^4^ cells/well and incubated overnight at 37°C with 5% CO₂. Staining after 24 hours of co-incubation with and 278 mM D-galactose (D-Gal) in human AC16 cells.


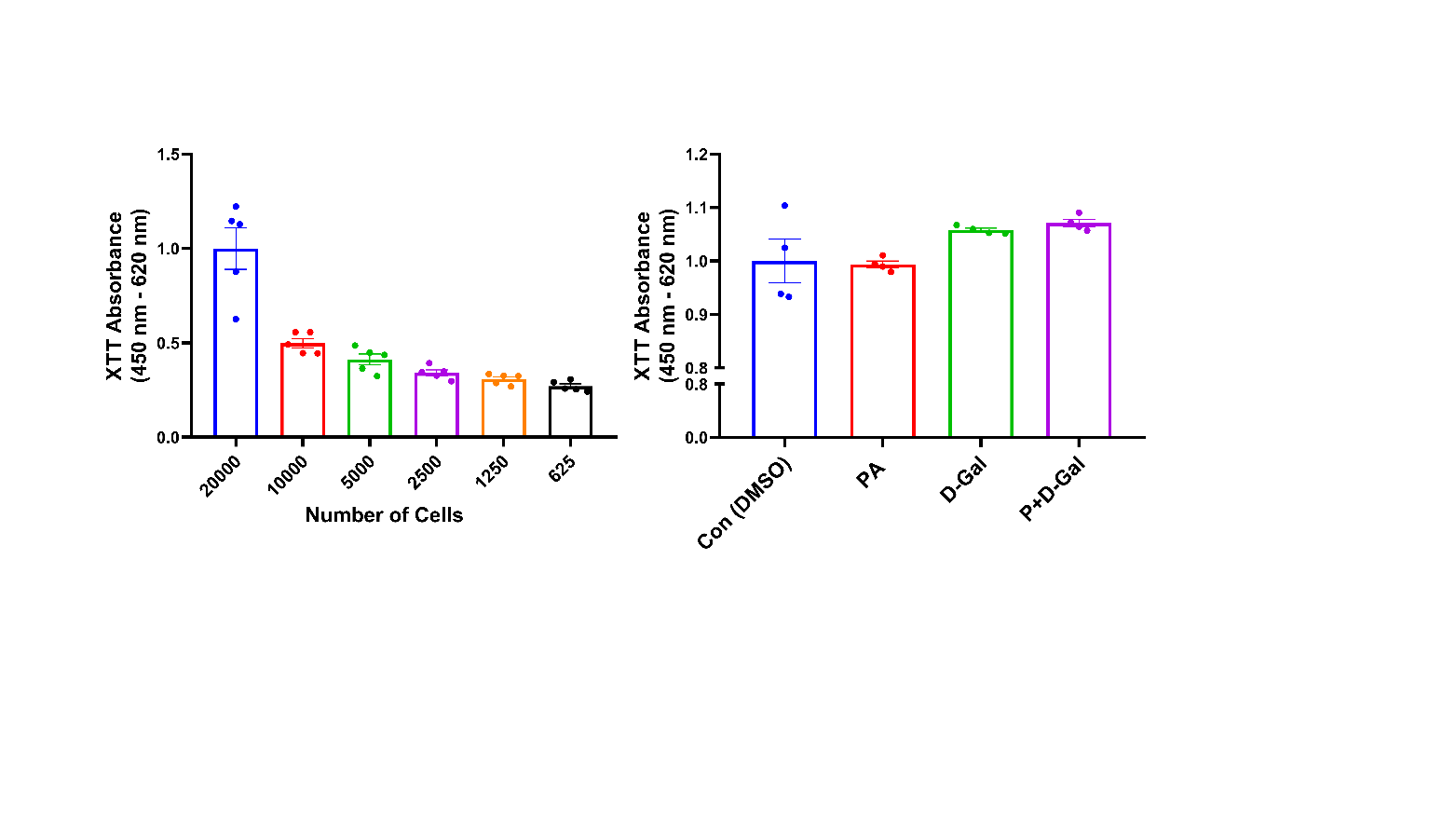
Cells were treated with bovine serum albumin conjugated palmitic acid (PA; 50 µM) and 278 mM D-galactose (D-Gal; 50 mg/ml) and 1/10000 DMSO vehicle, for 24 hours. After treatment, 0.3 mg/mL of XTT (0.63 mM) reagent, prepared per the manufacturer’s instructions, was added to each well. Plates were incubated for 4-5 hours, and absorbance was measured at 470 nm with a 650 nm reference using a microplate reader (1). Experiments were performed in quadruplicate (n=4), and data are expressed as mean ± SEM.

**Supplementary Figure 1**. Assessment of cell viability using the XTT assay after 24-hour incubation. (A) The dynamic range of the XTT measurements, illustrating the correlation between cell number and XTT absorbance, underscoring the assay's sensitivity and reliability. (B) XTT absorbance levels summarized for experimental groups. Data were presented as mean±SEM (n = 4-5 replicates per group).

**Senescence-associated β-galactosidase (SA-β-gal) staining**

Confirmation of cellular aging was conducted using the Senescence β-Galactosidase Staining Kit (Cell Signaling Technology; Cat: 9860), following the manufacturer’s protocol as outlined in previous studies (2). Cells were cultured in 6-well plates, treated according to the experimental design, and fixed with the provided Fixative Solution for 10–15 minutes at room temperature after rinsing with PBS. The staining solution was prepared by mixing the X-gal reagent with the staining buffer according to the kit instructions, ensuring a pH of 6.0 for selective detection of senescence-associated β-galactosidase activity. The prepared solution was added to the cells, and the plate was incubated at 37°C in a CO₂-free humidified chamber for 12–16 hours. Following incubation, cells were examined under a light microscope for blue staining, indicative of SA-β-gal activity and cellular senescence.


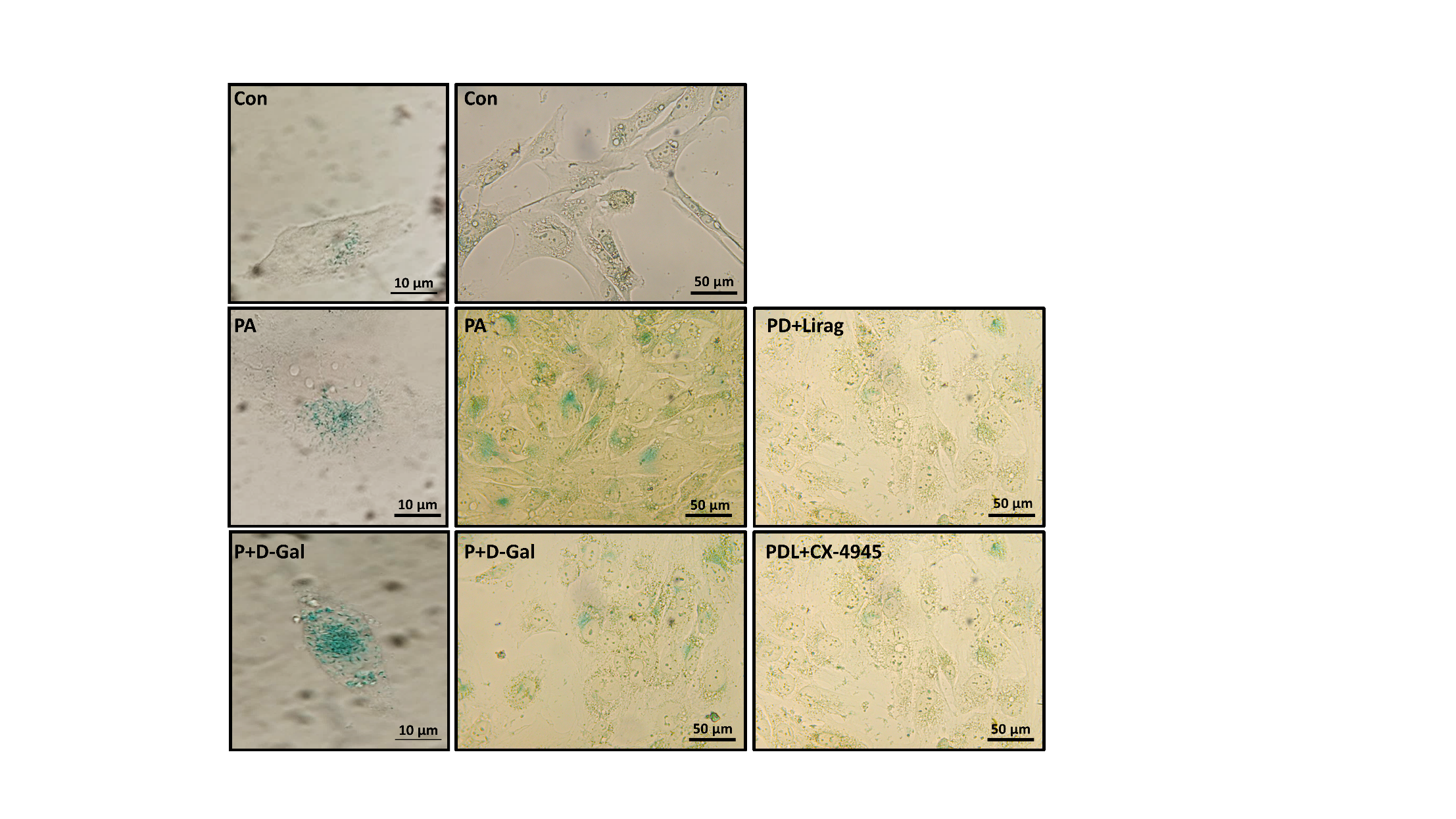
**Supplementary Figure 2.** **Characterization of the senescence-associated β-galactosidase (SA-β-Gal) staining in AC-16 cells.**  Representative images of increased SA-β-Gal staining is observed in cells incubated with PA and even more pronounced in a combination of palmitic acid with D-galactose (P+D-Gal) for 24 hours, indicating an enhanced level of cellular senescence. Chronic treatment with liraglutide and CK-2 attenuates senescence-associated β-galactosidase staining. The scale bars represent 10-50 µm.

**Reverse transcription-quantitative PCR (RT-qPCR)**

To assess mRNA levels, total RNA was extracted from the AC16 cells using the PureZOL™ RNA Isolation Reagent (7326880, Bio-Rad). RNA quality and concentration were assessed by electrophoresis on 1.5% Agarose gel (Fig. S1) and by nanodrop spectrophotometer (Epoch) (Supplementary Table 1). 1ug of RNA was reverse transcribed using the iScript™ cDNA Synthesis Kit (1708891, Bio-Rad). Quantitative PCR (qPCR) was performed in a Roche LightCycler 480 II system with SsoAdvanced™ Universal SYBR® Green Supermix (1725271, Bio-Rad). Fold changes in gene expression were determined using the comparative (2−ΔΔCt) method, with the untreated AC16 control group serving as the calibrator and Gapdh as the housekeeping control. Primers are listed in Supplementary Table 2.


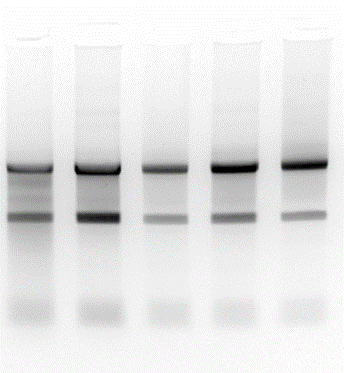


**Supplementary Figure 2**. Denaturing Agarose Gel Electrophoresis. Agarose gel electrophoresis of representative RNA samples derived from the treated AC16 cells (PD-Lirag, Lane1; P+D-Gal, Lane2; Con, Lane3; PA, Lane4; PDL+CX-4945 Lane 5). The integrity of the RNA is indicated by the presence of two bands corresponding to the 28S and 18S ribosomal subunits.

**Supplementary Table 1. Measurement of RNA sample concentrations and OD_260_/OD_280_ ratios using the Epoch microplate spectrophotometer.**

| **Name** | **260 Raw** | **280 Raw** | **320 Raw** | **260** | **280** | **260/280** | **ng/µL** |
| --- | --- | --- | --- | --- | --- | --- | --- |
| PD-Lirag | 0,66 | 0,371 | 0,073 | 0,579 | 0,293 | 1,974 | 463,296 |
| P+D-Gal | 0,784 | 0,417 | 0,043 | 0,736 | 0,371 | 1,986 | 588,88 |
| Con | 0,959 | 0,507 | 0,048 | 0,902 | 0,454 | 1,988 | 721,625 |
| PA | 1,135 | 0,587 | 0,046 | 1,083 | 0,537 | 2,015 | 866,068 |
| PDL+CX-4945 | 0,662 | 0,358 | 0,043 | 0,613 | 0,311 | 1,971 | 490,513 |

Primers are listed in Supplementary Table 2.

**Supplementary Table 2. Targeted mRNA Sequences and Primer Pairs Used for qPCR**

| Target mRNA | Forward Primer | Reverse Primer |
| --- | --- | --- |
| GRP78 | GCTGGAACTATTGCTGGCCT | CCCCCTCCCTCTTATCCAGG |
| Calnexin | AAGAGGCCACAAAACCCGAA | AGGAGCCTCCCATTCTCCAT |
| IRE-1 | GCCGAAGTTCAGATGGAATC | ATCTGCAAAGGCCGATGA |
| ATF4 | CACCGCAACATGACCGAAAT | GACTGACCAACCCATCCACA |
| ATF6 | GCTTTACATTCCTCCACCTCCTTG | ATTTGAGCCCTGTTCCAGAGCAC |
| CHOP | TTCTCTGGCTTGGCTGACTG | CTGCGTATGTGGGATTGAGG |
| p62 | TACGACTTGTGTAGCGTCTG | CGTGTTTCACCTTCCGGAG |
| LC3 | CGGAGAAGACCTTCAAGCAG | CTGGGAGGCATAGACCATGT |
| LONP1 | TCCTAAGCGGCTGTACAAGG | ACTTACGGTGGGTCTGCTTG |
| HSP10 | CTCCCAGAATATGGAGGCACC | TGGAATGGGCAGCATCATGT |

1. Kondo, T., Wada, K., Kawashima, M., Sato, Y. and Yamauchi, M. (1994). High-sensitivity antitumor drug sensitivity testing. Oncology 51: 535-539.

2. Debacq-Chainiaux, F., Erusalimsky, J.D., Campisi, J. and Toussaint, O. (2009). Protocols to detect senescence-associated beta-galactosidase (SA-βgal) activity, a biomarker of senescent cells in culture and in vivo. Nature protocols 4: 1798-1806.
